# Huitlacoche (*Ustilago maydis*), an Iconic Mexican Fungal Resource: Biocultural Importance, Nutritional Content, Bioactive Compounds, and Potential Biotechnological Applications

**DOI:** 10.3390/molecules28114415

**Published:** 2023-05-29

**Authors:** Zuamí Villagrán, Magdalena Martínez-Reyes, Horacio Gómez-Rodríguez, Uzziel Ríos-García, Efigenia Montalvo-González, Rosa Isela Ortiz-Basurto, Luis Miguel Anaya-Esparza, Jesús Pérez-Moreno

**Affiliations:** 1 Centro Universitario de los Altos, Universidad de Guadalajara, Tepatitlán de Morelos 47620, Mexico; 2 Edafología, Campus Montecillo, Colegio de Postgraduados, Texcoco 56230, Mexico; 3 Laboratorio Integral de Investigación en Alimentos, Tecnológico Nacional de México/Instituto Tecnológico de Tepic, Tepic 63175, Mexico

**Keywords:** edible fungi, food security, food diversification, biological activities, health benefits, genetic resource, mycochemistry, mycochemical profile

## Abstract

Worldwide, the fungus known as huitlacoche (*Ustilago maydis* (DC.) Corda) is a phytopathogen of maize plants that causes important economic losses in different countries. Conversely, it is an iconic edible fungus of Mexican culture and cuisine, and it has high commercial value in the domestic market, though recently there has been a growing interest in the international market. Huitlacoche is an excellent source of nutritional compounds such as protein, dietary fiber, fatty acids, minerals, and vitamins. It is also an important source of bioactive compounds with health-enhancing properties. Furthermore, scientific evidence shows that extracts or compounds isolated from huitlacoche have antioxidant, antimicrobial, anti-inflammatory, antimutagenic, antiplatelet, and dopaminergic properties. Additionally, the technological uses of huitlacoche include stabilizing and capping agents for inorganic nanoparticle synthesis, removing heavy metals from aqueous media, having biocontrol properties for wine production, and containing biosurfactant compounds and enzymes with potential industrial applications. Furthermore, huitlacoche has been used as a functional ingredient to develop foods with potential health-promoting benefits. The present review focuses on the biocultural importance, nutritional content, and phytochemical profile of huitlacoche and its related biological properties as a strategy to contribute to global food security through food diversification; moreover, the biotechnological uses of huitlacoche are also discussed with the aim of contributing to the use, propagation, and conservation of this valuable but overlooked fungal resource.

## 1. Introduction

**Figure 1:**
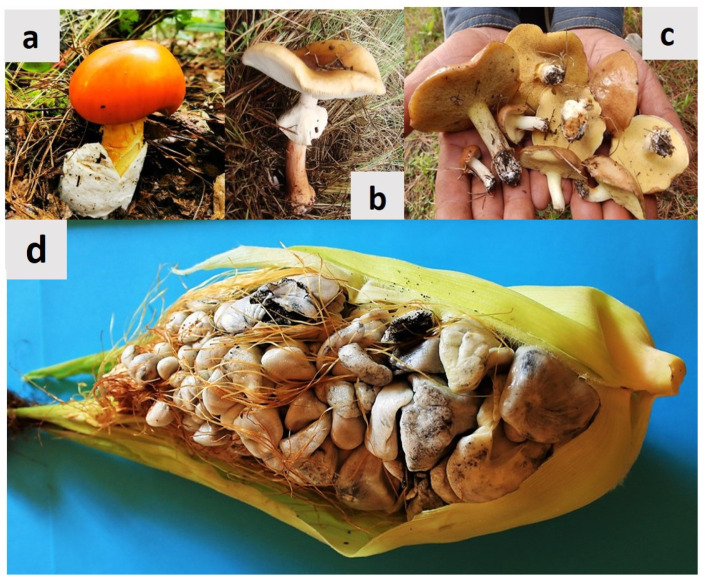
Biocultural diversity of edible wild mushrooms from Mexico. (**a**) *Amanita basii*, belonging to the Caesar’s mushroom group, which has an important international market; (**b**) *Amanita rubescens*, which is widely consumed throughout the country; (**c**) *Suillus luteus* from Mexico, commercialized mainly from South America, where it is an economically important non-timber forest product; (**d**) Huitlacoche (*Ustilago maydis*), a fungus that infects corn ears, and it is widely used in traditional Mexican medicine to heal more than 50 illnesses.

Fungi comprise the second-largest group of living organisms on Earth, and some of them are edible and have been consumed as food by humans since ancestral times [1,2]. In this context, due to their nutritive value and potential health benefits, the international trade of edible fungi has grown rapidly in both local and international markets in recent years [3,4]. The most expensive edible wild fungi are included in the genera *Cantharellus*, *Tuber*, *Tricholoma*, and *Boletus*, whose international annual in-season retail market ranges from USD 150 million to USD 1.67 billion [1]. Currently, 2189 species of edible fungus have been reported worldwide; after China, Mexico ranks second in terms of the biocultural heritage of edible fungus, where 450 fungal species are consumed as food, including a wide consumption and commercialization of *Ustilago maydis* (DC.) Corda [1] (Figure 1).

*U. maydis* belongs to the Ustilaginaceae family, and it is a basidiomycete fungus capable of infecting maize plants (*Zea mays* L.), producing galls on the ears of corn [5]. When young, it is light grey, but as it matures, it turns to a black color [5,6]. Around the world, *U. maydis* is considered a phytopathogen that causes severe damage to maize crops, leading to serious economic losses [6]. In this context, most research worldwide focuses on how to prevent its spread or control it [7]. By contrast, in Mexico, *U. maydis* is considered a delicacy that is consumed by 21 ethnic groups, and it is known by a variety of names in traditional languages (Table 1), which denotes its ancestral use. Due to the fact that this fungus is only consumed traditionally in Mexico, and in no other country, it is considered an icon of the national cuisine [8,9] and an alternative crop with agro-alimentary importance due to its nutritive value [10]. Additionally, in Mexico, it is used to heal 55 different diseases, being the fungal species with the greatest importance in traditional Mexican medicine [11,12,13,14]. The therapeutic effects of *U. maydis* are attributed to bioactive molecules that exert beneficial physiological effects on human health [15].

Due to the unequal economic distribution among human societies, one of the most critical problems to be solved is feeding a constantly growing population, where edible fungus, including huitlacoche, could play an important role in food security due to its nutritional value and nutraceutical potential [1,3]. On the other hand, agriculture in Mexico is marginal and collapsing [41]; thus, one way to encourage the younger generations in the countryside is to provide jobs and sources of income by cultivating huitlacoche [42], which involves not only economic benefits but also nutritional, health, social, and cultural aspects [2]. The present review focuses on the biocultural importance, nutritional content, and phytochemical profile of huitlacoche and its related biological properties as a strategy to contribute to global food security through food diversification; moreover, the biotechnological uses of huitlacoche and its relevance in food security and sustainable development are also discussed, with the aim of contributing to the use, propagation, revalorization, and conservation of this valuable fungal resource.

## 2. Huitlacoche (*Ustilago maydis*)

*Ustilago maydis* is known as “black mold”, “Mexican truffle”, “cuitlacoche”, or “huitlacoche”. It is a biotrophic and ubiquitous phytopathogenic fungus that belongs to the *Ustilaginaceae* family [43,44]. This basidiomycete fungus is the causal organism of corn smut disease (Figure 1e), which leads to severe damage in corn plants [45,46] and is a parasite exclusive to this crop and its predecessor, teosinte (Teocintle, *Zea* spp.) [7]. The most evident signs that the corn smut disease produces are tumors with dark diploid teliospores [47], mainly on corn ears, but it also can infect all maize plant parts including stems, leaves, and tassels [48]; the corn plant can be infected by this fungus in all phenological phases [49] due to the ability of *U. maydis* to switch from the yeast-like form (non-pathogenic) to the filamentous cell form (pathogenic) [50]. In this context, *U. maydis* has been the focus of extensive research (genetic regulation, mechanisms of pathogenicity, and relation with the host) because it is considered a model phytopathogen [7,45].

To colonize its host, *U. maydis* has developed diverse biological strategies based on the secretion of effectors (proteins) that mediate its interaction with maize [51], by reprograming or altering the host metabolism and facilitating the fungal infestation, expansion, and colonization [7]. The initial steps of *U. maydis* tumor formation are characterized by hypertrophy (substantial growth) and hyperplasia (excess host cell division) during the first five days of infection; nonetheless, complete plant fungal infection usually occurs after 15 days post-infection; at this point, the tumors are mature and could repeat the life cycle of *U. maydis* by the release of diploid teliospores [51]. The main control strategies used to avoid *U. maydis* infection in maize crops in many countries are based on the use of fungicides, biological control, modification of fertility, crop rotation, seed treatment, and cultivation of resistant varieties and genetic improvement, which are the most effective when used with maize-resistant varieties [52,53].

**Figure 2:**
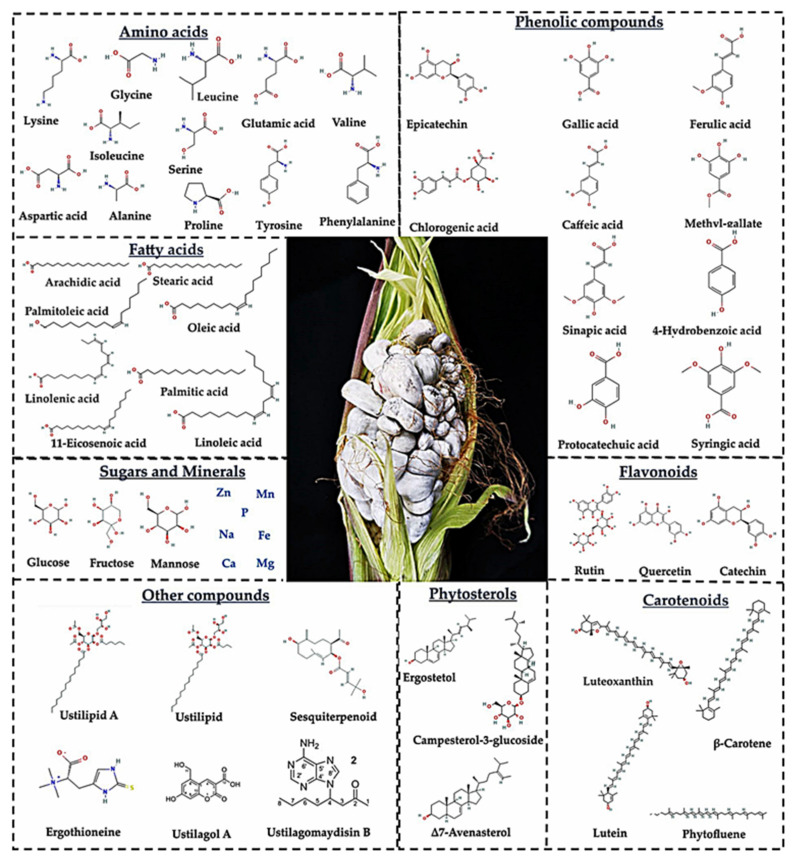
Nutritional and phytochemical compounds reported in huitlacoche.

Despite *U. maydis* being considered a maize pathogen and the fact its presence is undesirable in most maize-growing regions globally [51], in Mexico it is considered an economically important fungal resource [54,55] that represents an alternative crop and a delicacy [56,57]. Huitlacoche is a perishable edible fungus that, if grown traditionally, is only available usually for two months (July and August) [48]. However, the cultivation of this fungus, infecting the early stages of corn ears called “*jilote*” in Spanish, has already been achieved [58,59,60]. Furthermore, it has been reported that huitlacoche as a crop can be more profitable than maize itself, increasing the value of maize cultivars by 20 to 50 times their market price [61,62]. Additionally, huitlacoche is a reliable source of nutritional components and phytochemicals (Figure 2), which can be exploited in diverse industrial applications [6,55,63,64,65].

### 2.1. Literature Search Strategy and Bibliometric Analysis on Ustilago maydis

In order to evidence the current knowledge, scientific perspectives, and potential technological applications of huitlacoche as a food source, a bibliometric analysis was performed. For this purpose, in February 2023, a bibliometric search divided into two steps was conducted in the Scopus database. The search pattern TITLE-ABS-KEY was “*Ustilago maydis*” for the first bibliometric search. In addition, the term “huitlacoche” was used in a second search. All searches were limited to original articles published between 2000 and 2022, where review articles, books or book chapters, short surveys, conference papers, editorials, notes, and letters were excluded. Following the exclusion step, distribution data by year, geographic area (country/territory), funding sponsor, publication area, and languages were extracted directly from Scopus. Then, the data collected in Scopus were analyzed using the VOSviewer software (version 1.6.16), exploring the distribution and connection of searching terms.

#### Huitlacoche (*U. maydis*) Bibliometric Analysis

**Figure 3:**
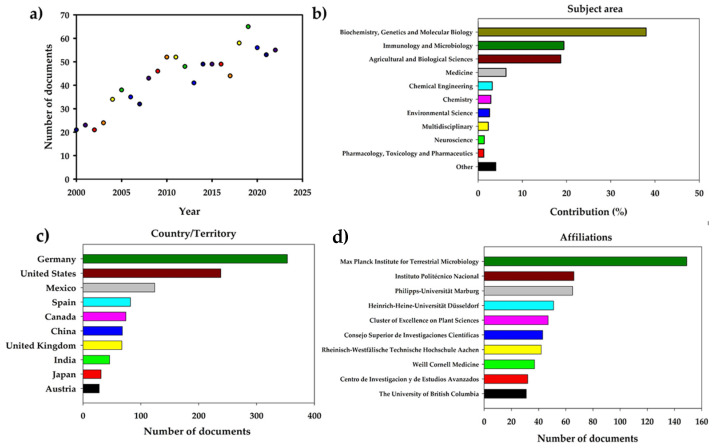
Bibliometric analysis on *Ustilago maydis* research: (**a**) documents published by year, (**b**) subject area of publication, (**c**) country of publication, (**d**) affiliations of the authors.

The first search revealed 989 articles on *Ustilago maydis*, 969 of which were written in English, 8 in Chinese, 5 in German, 4 in Spanish, 1 in Hungarian, 1 in Polish, and 1 in Russian. The second search showed 26 documents on huitlacoche—24 written in English and 2 in Spanish. Furthermore, the highest concentration of articles was published in 2019, with 65 research articles. The number of publications has grown over time (Figure 3a). Articles related to *U. maydis* were published in 21 subject areas (Figure 3b), where the most representative areas were Biochemistry, Genetics, and Molecular Biology (38%); Immunology and Microbiology (19.4%); and Agricultural and Biological Sciences (18.7%). In this context, Germany is the country with the highest contribution on *U. maydis* (353 documents), followed by the United States (238 documents), and Mexico (124 documents), as shown in Figure 3c. These studies were conducted mainly by researchers affiliated to German institutions, such as Max Planck Institute for Terrestrial Microbiology (149 documents), Philipps-Universität Marburg (65 documents), Heinrich-Heine-Universität Düsseldorf (65 documents), the Mexican Instituto Politécnico Nacional (IPN) (66 documents), and the Centro de Investigación y de Estudios Avanzados, IPN Campus Guanajuato (32 documents) (Figure 3d), which were sponsored mainly by the Deutsche Forschungsgemeinschaft in Germany and the Consejo Nacional de Ciencia y Tecnología in Mexico.

Generally, *U. maydis* is described as a maize pathogen in the literature. Most research centers have focused their attention on searching for alternatives to its control, primarily through molecular studies that include gene expression, signal transduction, dimorphism, pathogenicity, and disease development [7]. Regarding articles published by Mexican authors, publications also contain molecular studies related to *U. maydis* aiming to develop strategies for increasing huitlacoche yield, and diverse dynamics to harvest this fungal resource have also been evaluated [56,61,66,67]. Nonetheless, Mexican research is also focused on the nutritional, physicochemical, thermal, and rheological characterization of huitlacoche powder and its use in the development of functional foods [8,68,69,70], as well as the identification of bioactive compounds and their potential biological activities for pharmaceutical applications [6,55,63,64,65].

**Figure 4:**
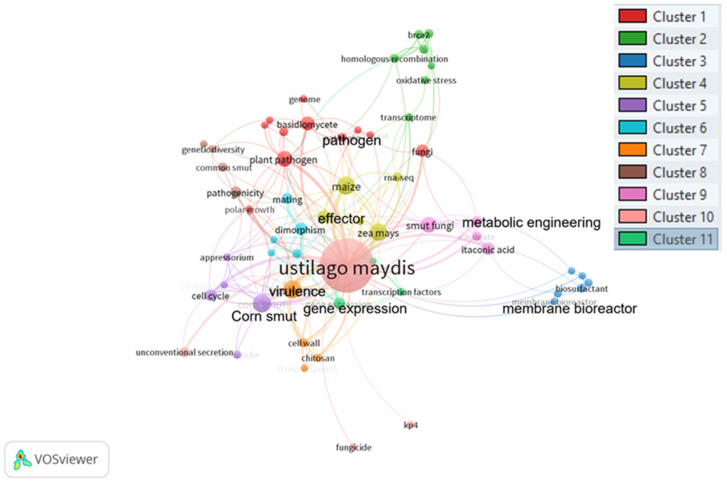
Distribution of searching terms on articles published on *Ustilago maydis* from 2000 to 2022. Cluster 1: plant pathogen; Cluster 2: transcriptomic studies; Cluster 3: biosurfactant; Cluster 4: maize effector; Cluster 5: metabolic engineering; Cluster 6: dimorphism; Cluster 7: cell wall; Cluster 8: pathogenicity; Cluster 9: fungicide; Cluster 10: *Ustilago maydis*; Cluster 11: gene expression. Figure created with VOSviewer software version 1.6.16. The results are based on the threshold of 73 terms (from 2187 keywords) with at least 5 co-occurrences.

Figure 4 shows keyword co-occurrence in articles published in the Scopus database related to *Ustilago maydis* during the last 22 years. It can be observed that the distribution of terms is centered in 11 clusters around the *U. maydis* term. Based on these findings, most research focuses on identifying *U. maydis* gene expression, pathogenicity on maize crops, cell cycle, and control strategies. On the other hand, some documents are centered on the metabolic engineering of the fungus in biotech factories, to produce itaconic acid. The bibliometric analysis results provided information on publication trends, demonstrating the primary interests in the *U. maydis* research. Conversely, although the bibliometric analysis did not reveal any potential biotechnological applications of *U. maydis* (except for itaconic acid production), it should be noted that this fungal resource has a wide range of technological applications and is a valuable natural resource with high nutritional and nutraceutical value, as discussed below.

## 3. Biocultural and Gastronomic Importance of Huitlacoche

Mexico is a biologically diverse country characterized by its culture and traditional knowledge of gastronomy and medicine [71]. According to Molina-Castillo et al. [72], the Mexican food system could be classified into three categories: central (consumed every day, such as maize, beans, and chilies), secondary (consumed frequently, such as meat, eggs, potatoes, and tomatoes), and peripheral (consumed only during seasonal periods). In this context, Mexican cuisine is distinguished by including wild foods such as edible fungi based on their traditional knowledge and practice, particularly huitlacoche, consumed predominantly in the rural population [73,74]. Nonetheless, *U. maydis* and maize exhibited a related co-evolution associated with maize domestication and cultivation throughout the Americas, mainly in Mexico, which is considered the center of origin of maize but also where the Mexican diet is highly centered on corn-based products [67,75].

**Figure 5:**
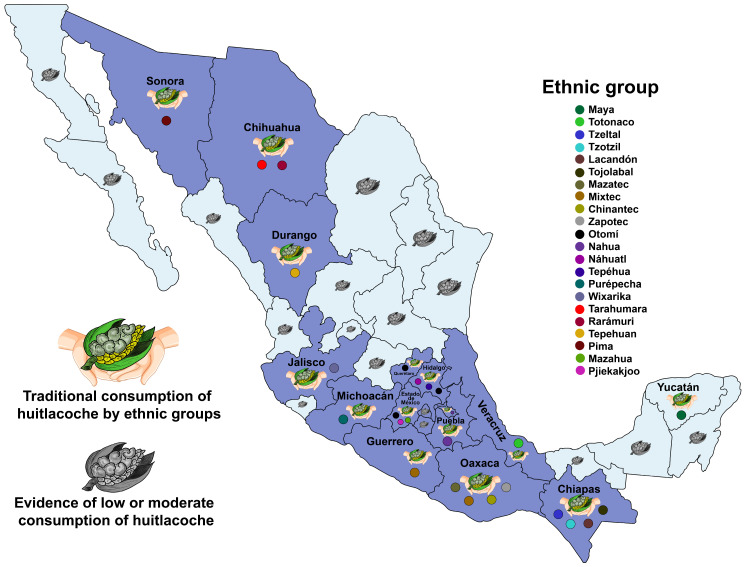
Ethnic groups that give a traditional name to huitlacoche fungus in Mexico, and consumption distribution.

The consumption of huitlacoche is endemic to Mexico [71,76]; its Nahuatl name (the language of Aztec civilizations) is derived from *cuitla* (excrement) and *cochi* (pig), which means pig’s excrement [77]. Currently, it is consumed and has been given numerous autochthonous names by more than 20 ancient Mexican ethnic groups, distributed mainly in Central and Southeastern Mexico but also in the north of the country [18], as listed in Table 1. In the Mixtec group (the third-largest indigen group after Nahua and Maya), it is called *tɨká maa* (*tɨka* = grasshopper; *maa* = bad), which means bad grasshopper [24]; meanwhile, it is called *ta chak* by the Mayas [78]. In some communities in Zacatecas, huitlacoche is called *coloche* or *pitacoche* [79]. In the Michoacan state (Purepecha culture), it is called *terékua* (means mushroom in Purepecha language), while it is called *kjú tha* in Otomí (Mexico state) and *sunó weko wiwara* in Rarámuri culture in the Chihuahua state [18]. In the Yucatan state (Maya culture), *U. maydis* is highly appreciated as food [80]. Nonetheless, in some Mayan and mestizo communities, this edible fungus is recollected for consumption within the family unit and is not collected for sale [18]. Additionally, the Wixarika culture (located in the northern region of Jalisco state) consumes huitlacoche (named *Ki’au*) as a food or ceremonial drink (*tsinari* or atole negro) [71]. Furthermore, huitlacoche is one of the most important edible fungi with cultural significance, nutritional value, and health benefits in one community of San Mateo Huexoyucan in the Tlaxcala state [24]. In the Chiapas state, it is used to prepare *smoloc*, a cold beverage, while in the Oaxaca state it is used to make “Mole negro” [81]. Presently, in Mexico, the consumption of huitlacoche is widely distributed, mainly in the center and southeast of the country (Figure 5).

**Figure 6:**
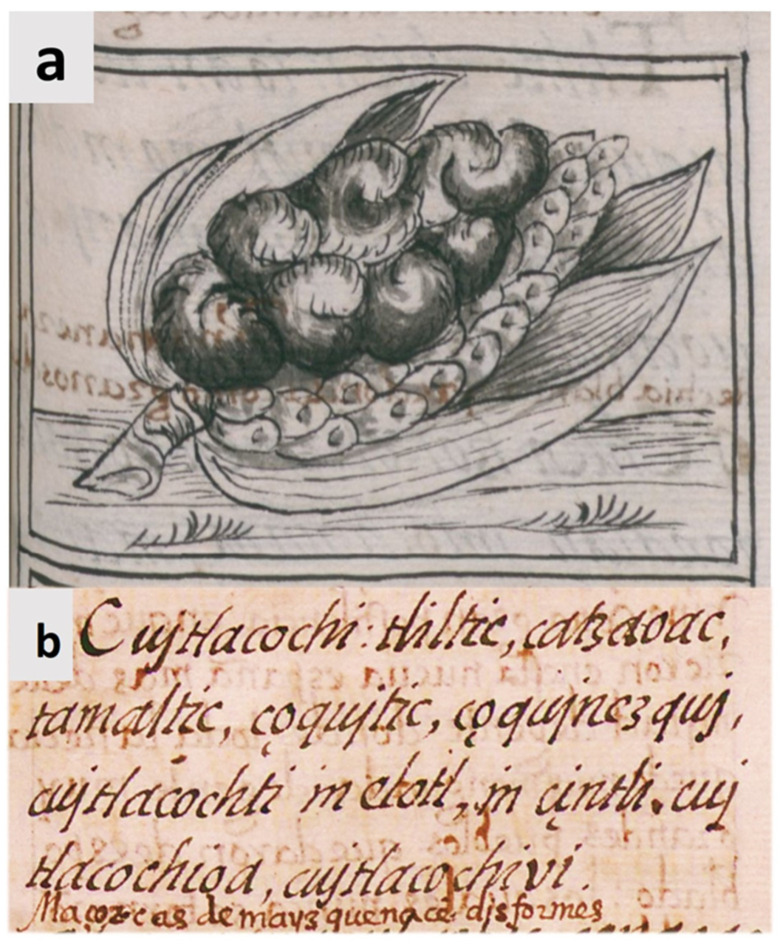
The most ancient evidence of huitlacoche in Mexico recorded in the Florentine Codex, dating from the mid-XVI century. (**a**) A figure of huitlacoche drawn by Aztec people, which appeared in Book 11, folio 251 of the Florentine Codex; (**b**) Description of the huitlacoche, called *cujtlacochi*, in the Nahuatl language, which appears in Book 11, folio 251 of the Florentine Codex.

The origin of the consumption of huitlacoche as food has long been a matter of debate [36], since the documentary evidence is scarce, so it is reasonable to affirm that the beginning of its consumption is lost in the mists of time. One of the reasons for this fact is the enormous destruction of the pre-Hispanic codices after the conquest of Mexico by the Spaniards and the lack of knowledge related to the ethnic groups that inhabit the north of the country. However, the first documented evidence of the knowledge of this fungus in Mexico is found in the Florentine Codex, which dates back to the mid-16th century. In this Codex, two important pieces of evidence can be appreciated: (i) the first is an illustration of the fungus infecting a corn cob (Figure 6a); and (ii) the second is a detailed description in Nahuatl, the language of the Aztecs, which literally says “… *Ear of corn that is born deformed, Cujtlacochi, it is black, dark, like a tamal (*a traditional mexican dish*), it looks like mud, it appears like mud. On green ears, on ripe ears it becomes ash, forms ash, turns ash*…” (Figure 6b).

**Figure 7:**
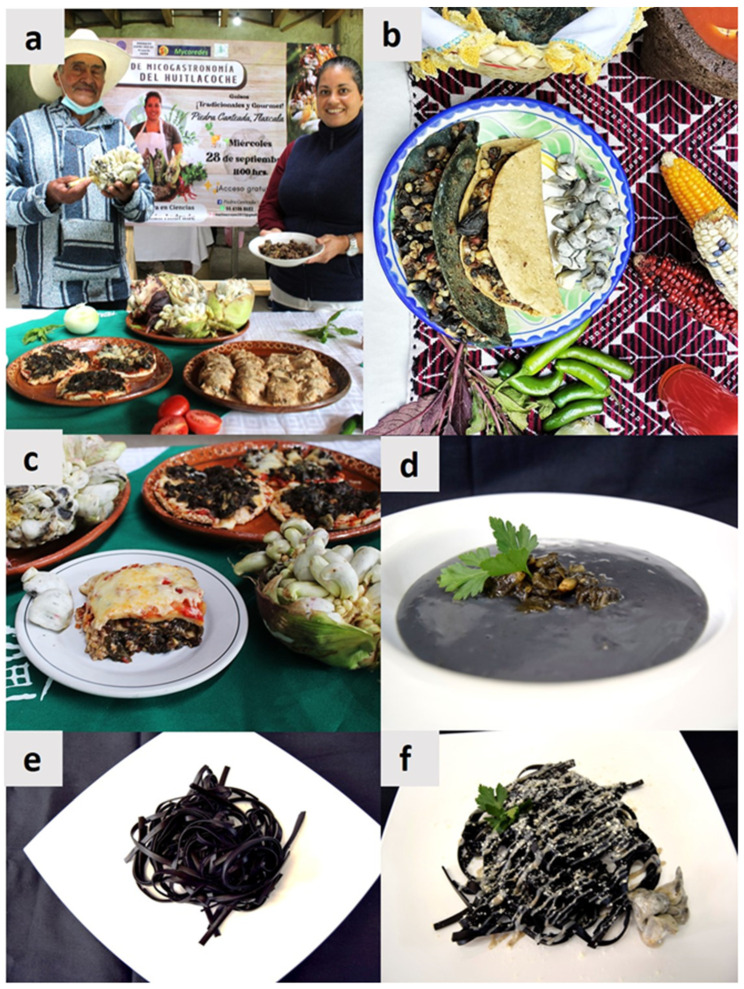
Mexican mycogastronomy of huitlacoche: (**a**–**c**) Traditional dishes; (**d**–**f**) Gourmet dishes; (**a**) Course of gastronomy of huitlacoche by a Mexican chef, taught to peasants in Piedra Canteada, Tlaxcala, in Central Mexico; (**b**) One of the most common dishes in which huitlacoche is consumed is called “quesadillas” in Spanish; (**c**) Different dishes using huitlacoche as the main ingredient; (**d**) Huitlacoche cream; (**e**) Fettuccini pasta containing huitlacoche; (**f**) Fettuccini gourmet dish containing huitlacoche.

Huitlacoche is popular in Mexican cuisine because of its exotic flavor, which is acidic, astringent, earthy, bitter, and umami [58]. It is considered a delicacy and is used in a wide variety of food dishes (Figure 7a–f), including “*antojitos mexicanos*” as “quesadillas, tacos, tlacoyos, huaraches, sopes, enchiladas”; moreover, huitlacoche has been incorporated into modern food products such as soups, pasta, pizza, and bakes, among other things [68,72,82]. However, the most common way to prepare huitlacoche is by cooking it in a stir-fry in oil with onion, garlic, chili pepper, and epazote [81]. Huitlacoche recipes can be found online and in some Mexican cookbooks [15].

**Figure 8:**
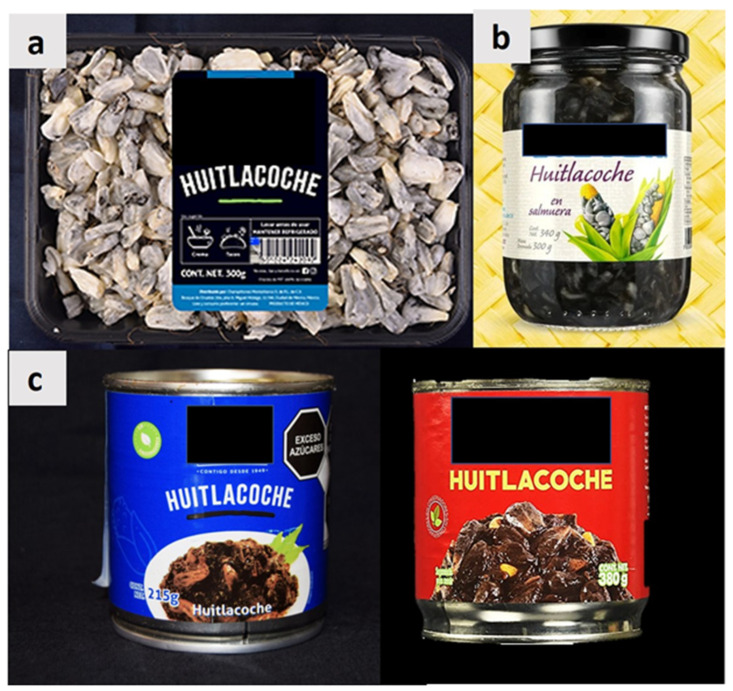
Examples of different huitlacoche products commercialized in Mexico: (**a**) refrigerated galls; (**b**) brine in bottle; (**c**) canned products.

Furthermore, huitlacoche can be sold fresh, canned (with or without other vegetables), or as a lyophilized/dehydrated product on the market (Figure 8a–c) [15]. In this context, using huitlacoche in food products with potential functional properties may be a viable alternative to its valorization, changing the perception of this natural resource as a corn pest [83]. In this context, its consumption has drawn a recent increasing interest worldwide (e.g., in Latin America, the United States, Japan, and Turkey) as a gourmet food [11,73].

On the other hand, its ancestral medicinal use presents a different scenario, compared to that of its use as food. It can be affirmed that in Mexico it has been widely used since pre-Hispanic times by a large number of native cultures as medicine. Of the 200 species of medicinal mushrooms known in Mexico, the huitlacoche is the mushroom used most in traditional Mexican medicine. It is used to heal 55 illnesses in various ethnic groups, including: heart disease, colic, blisters, pimples, skin burns, athlete’s foot, wounds, nosebleeds, baby rashes, stopping hemorrhages, healing animal bites, alleviating dehydration, and helping with anxiety, as well as to treat diarrhea, indigestion, intestinal pains, and inflammations [11,12,13,14,84]. These beneficial effects could be attributed to the presence of various secondary metabolites (organic acids, phenolic compounds, and carotenoids) and to the fiber content of huitlacoche, including β-glucans that exert prebiotic properties [15,69,85]. Additionally, it has been used by an Otomi group in the state of Tlaxcala in Central Mexico as a cosmetic to enhance female beauty because of its properties to soften and refresh the skin. This ethnic group mixes the spores of the fungus with lemon juice and applies it to the face as a mask [86].

### 3.1. Nutrimental Content

Huitlacoche plays an important role in Mexican gastronomic culture due to its traditional uses, sensory attributes, and nutritional value (Table 2) [6,71]. It contains adequate protein and soluble and insoluble dietary fiber contents, which have significant benefits for consumer nutrition and health [15]. Furthermore, huitlacoche contains β-glucans (20–120 mg/100 g), compounds classified as prebiotics that exhibit antidiabetic properties [87]. However, the nutritional composition of huitlacoche may be influenced by the type of maize used and the stage of development in which it is harvested [6,15,65]. On the other hand, according to the Mexican Equivalent Food System, 66 g of cooked huitlacoche contains only 20 kcal, making it a low-calorie food [88].

Additionally, huitlacoche contains amino acids, fatty acids, monosaccharides, oligosaccharides, and minerals [55,89], as shown in Table 3. In general, huitlacoche has almost all essential amino acids, being the most abundant in lysine, glycine, and leucine [15,89], and essential fatty acids including oleic and linoleic (precursors of omega 3 and omega 6), compounds with high nutritional value [15]. Furthermore, this fungal resource has carbohydrates that are easily digestible such as glucose and fructose [15,65], and minerals such as phosphorous, magnesium, and calcium [15], which have been shown to play an important role in bone health [90,91]. Additionally, it has been reported that raw and cooked huitlacoche provide vitamin A, B9, and C [66,88].

According to these data, huitlacoche consumption is a viable addition to the human diet [4], providing nutritionally important compounds that significantly contribute to the Sustainable Development Goals proposed by the United Nations (e.g., zero hunger and food security) through food diversification [92].

### 3.2. Mycochemical Compounds of Huitlacoche

Huitlacoche is an edible fungal resource containing many phytochemical compounds with potential biological properties and health benefits [6,8,15,64,65]. Identified compounds (quantitative or qualitative) include polyphenols, flavonoids, carotenoids, phytosterols, purine-derived, and terpenoids, among others (Table 4).

Phenolic, flavonoids, and carotenoids are recognized as antioxidant compounds that exhibit potential health benefits and pharmaceutical and food industrial applications. The main phenolic compounds reported in huitlacoche include ferulic (358 µg/g), sinapic (36 µg/g), chlorogenic (15.94 µg/g), *p-*coumaric (12 µg/g), and caffeic (11.2 µg/g) acids [6,8,15,64,65]. Furthermore, the presence of flavonoids such as anthocyanins (89.8–226 mg/kg cyanidin-3-glucoside), quercetin (33 µg/g), naringenin (14 µg/g), catechin (10–11 µg/g), and rutin (5 µg/g) [15,63,64,65] has been reported, while β-Carotene (15 µg/g) and β-Cryptoxanthin (1.13 µg/g) are the most representative carotenoid compounds reported in huitlacoche [65,85]. Evidence suggests that huitlacoche is high in antioxidant compounds; nonetheless, phytochemical-rich diets have been linked to a risk reduction of non-transmittable diseases, due to the ability of these compounds to mitigate oxidative stress [97]. Therefore, huitlacoche consumption can improve human health status and prevent non-communicable chronic diseases.

Phytosterols are compounds analogous to cholesterol. They exhibit human health benefits due to their antioxidant and cholesterol-lowering properties. These compounds are commonly found in fruits, vegetables, nuts, legumes, whole grains, tubers, sunflower seeds, and vegetable oils [98]. The presence of phytosterols such as Campesterol-3-β-glucoside (8.25–12.94 µg/g), Δ7-stigmasterol (4.25–5.92 µg/g), Δ7-avenasterol (3.83–5.81 µg/g), and ergosterol (3.24–4.19 µg/g) have been reported in huitlacoche [64]. In this context, the consumption of this edible mushroom might contribute to the recommended daily intake of phytosterols [99].

Other bioactive compounds reported in huitlacoche include Ustilagol, Ustilagomaydisin, ergothioneine, sesquiterpenes, and ustilipids; these compounds exhibited interesting biological properties with pharmaceutical properties. Ustilagols A–F are coumarin-derived compounds obtained after huitlacoche fermentation that, in in vitro studies, exhibited potent anti-inflammatory and antithrombotic properties [13]. Furthermore, Ustilagomaydisins A–C are purine-derived compounds isolated from ethanolic extracts of huitlacoche; these compounds showed cytotoxic activities against multidrug-resistant human leukemia cells (K562/A02) at low doses [93]. Ergothioneine is an amino acid reported in huitlacoche with strong antioxidant properties [11,96]. Additionally, it has been reported that *U. maydis* can produce sesquiterpenoid compounds, which can be used as antimicrobial agents [100]; moreover, it has been reported that ustalipid A exerts dopamine effects in a concentration-dependent response [95].

According to these data, huitlacoche could be a reliable source of phytochemicals with beneficial health benefits that can be used to develop functional, nutraceutical, and pharmaceutical products.

### 3.3. Ustilago maydis as a Biotech Factory

Despite the fact that *U. maydis* has commonly been related to corn-plant infections, it is characterized by synthesizing intra- and extracellular compounds with potential biotechnological uses, including glycolipids, mannosylerythritol lipids, itaconic acid, siderophores, amino acid tryptophan-derived compounds, and hydrolytic enzymes [101,102,103].

It has been reported that *U. maydis* can produce glycolipid-type biosurfactants when grown in a medium with a limited nitrogen source [101]. Furthermore, *U. maydis* can convert crude glycerol into glycolipids [104]. Glycolipids could be used in the cosmetic, pharmaceutical, and food industries [105]. These compounds exhibited antioxidant properties and antimicrobial activity against other fungi and Gram-positive and Gram-negative bacteria [104,105,106]. Additionally, huitlacoche produces mannosylerythritol lipids (MELs), extracellular compounds that can serve as biosurfactants due to their amphipathic character [107]. These compounds exhibited potential for diverse biotechnological applications, mainly pharmaceutical, due to their dopaminergic and antimicrobial effects [108]. Moreover, MELs can be used to develop sustainable detergents and emulsifiers [94]. Additionally, depending on the carbon source, this basidiomycetous fungi can produce ustilagic acid C (a kind of MEL) that exhibits antimicrobial activities [108,109]; nonetheless, this compound can be used as a biocontrol agent of *Botrytis cinerea* due to its antagonistic effect [101].

Itaconic acid (IA, C_5_H_6_O_4_) is an organic acid that can be produced by fermenting lignocellulosic biomass with various fungi, including *U. maydis* [110]. IA is thus biodegradable when used as a monomer to manufacture polymers; it is non-toxic, and can be combined with other monomers to create a wide range of other polymeric derivatives [111]. The main application of IA focuses on developing synthetic latex, unsaturated polyester resins, superabsorbent polymers, chelant dispersant agents, biofuels, and methacrylate production [110]. In this context, several research studies have been conducted aimed at increasing the rate, yield, and purity of IA produced by *U. maydis* under submerged or solid-state fermentation processes [112,113,114] using different substrates (including agro-wastes), experimental conditions, biomass pre-treatments, and metabolic and morphological engineering modifications [50,112,115]. Previously, a yield of IA production has been reported to range from 12 to 34%, using glucose as substrate with a total productivity of up to 0.07 g/L/h [50,116]. However, the IA yield depends on the substrate composition, microbial strain, and the fermentation process [116]. Because these yields are not yet enough to be used for industrial purposes [50,112,113], further studies would be desirable in order to increase the yield of IA production using *U. maydis*. Furthermore, a consolidated bioprocess to produce IA by a co-culture of *U. maydis* and *Trichoderma reesei* was recently reported, achieving an efficient transformation of recalcitrant cellulose into IA [116].

Other secondary metabolites able to produce by *U. maydis* are siderophores ferrichrome and ferrichrome A; these compounds are cyclic peptides that exhibit iron-chelating properties [117] and could be used in agriculture, pharmacology, medicine, bioremediation, and the food industry [118]. Furthermore, *U. maydis* can synthesize indole pigments (compounds derived from the amino acid tryptophan), including those with a potential role in the treatment of pityriasis versicolor, a human skin disease [101,103].

Additionally, the ecological function that *U. maydis* performs in nature (degradation of lignocellulosic compounds) has given it access to a number of hydrolytic enzymes with promise for biotechnology, most of which act on polysaccharides [102]. A chlorogenic acid esterase from *U. maydis* has been purified, which can release *p-*coumaric, caffeic, and ferulic acids from complex lignocellulosic substrates [119]. Furthermore, the lipase UM03410 isolated from *U. maydis* showed trans-fatty acid selectivity; this makes this lipase a promising biocatalyst and valuable from a biotechnological perspective [120].

As a biological factory, *U. maydis* could be considered a perfect system with great potential for diverse biotechnological applications. However, further research is needed to increase this valuable compound’s yield recovery.

### 3.4. Potential Technological Applications of Huitlacoche

Several technological uses of huitlacoche have been associated with different bioactive molecules. These are antioxidants, the development of functional foods, the synthesis of inorganic nanoparticles, and some pharmaceutical and environmental applications, as discussed below.

#### 3.4.1. Antioxidant Capacity

Various reactive oxygen species (ROS), including hydroxyl radicals, hydroxyl ions, and superoxide anions, are created in nature, even in the human body. Therefore, to neutralize these reactive substances, the consumption of food rich in antioxidant compounds is recommended [11]. In this context, basic techniques for estimating the antioxidant capacity of food systems include ABTS, DPPH, FRAP, and ORAC [121]. In general, huitlacoche exhibited good antioxidant properties (Table 5). Nonetheless, multiple extraction methods (maceration, ultrasound-assisted, stirring, and shaking) and various solvents (ethanol and methanol either alone or combined with water) can be used to obtain antioxidant extracts from huitlacoche samples [6,8,15,63,64,65].

Ethanolic extracts from huitlacoche powder have shown to exhibit antioxidant activity by ABTS (200–312 mmol of trolox equivalents (TE)/mL), DPPH (30–165 mmol TE/mL) and FRAP (11–251 mmol TE/mL); nonetheless, it has been reported that the antioxidant activity of huitlacoche is increased after a cooking process, associated with the release of phenolic compounds from the food matrix [64]. Similar trends were reported in fettuccine pasta supplemented with huitlacoche powder [8]. It has been reported that the antioxidant activity of huitlacoche extracts (cultivated in maize creole genotypes) measured by DPPH correlates (*r* = 0.6461) with the phenolic content [15,63]. Moreover, glycolipids from huitlacoche also exert antioxidant activity in ABTS radical scavenging tests [106]. On the other hand, it must be considered that the antioxidant capacity of huitlacoche is affected by its stage of development [15,63], its geographic location of cultivation [65], its extraction procedure, and the solvent used [6].

The bioaccessibility of phenolic compounds from huitlacoche and their antioxidant capacity during gastrointestinal digestion (in vitro) has been previously evaluated; this parameter indicated the potential intestinal absorption of the bioactive compounds and their availability during oral, gastric, and intestinal digestion. In this context, undigested huitlacoche contains phenolic compounds with antioxidant properties, which was found using DPPH and ABTS tests [13.94 mg of gallic acid equivalents (GAE)/g, 12.51 mg TE/g, and 9.58 mg TE/g, respectively]. Moreover, these values were increased during oral (19.76 mg GAE/g, 61.33 mg TE/g, and 32.73 mg TE/g, respectively) and gastric phases (30.22 mg GAE/g, 31.29 mg TE/g, and 64.71 mg TE/g, respectively). On the other hand, at the end of the gastric phase, a decrease of phenolic compounds was observed (6.79 mg GAE/g), but antioxidant capacity showed increased values compared to undigested samples (DPPH = 25.51 mg TE/g and ABTS = 40.54 mg TE/g). These results demonstrate that the consumption of huitlacoche provide antioxidant compounds with beneficial effects to the human body [6].

Evidence suggests that huitlacoche is an excellent source of natural antioxidants important for dietary consideration since they can stop or prevent oxidative stress in human cells promoted by free radicals. In this context, these results support the folkloric use of huitlacoche in Mexican ethnic groups to treat some ailments and its potential use in developing functional foods and nutraceutical products.

#### 3.4.2. Development of Potential Functional Foods

Huitlacoche is an edible but highly perishable fungus (<3 days under ambient temperature); however, some strategies have been applied aimed at enhancing its shelf life [74]. In this context, huitlacoche has been explored as a functional ingredient to elaborate foods with potential health benefits in recent years [8,48,70]. The effect of huitlacoche flour addition on the functional and physicochemical properties of blue corn tortilla chips has been evaluated. An increase in total dietary fiber (↑175%), phenolic compounds (↑114%), and antioxidant capacity (↑18%) compared to the tortilla chip without huitlacoche-added flour has been found [70]. Moreover, the color of the tortilla chips was influenced by adding huitlacoche flour (black color) in a dose-dependent response. On the other hand, there was an increased breaking force as the huitlacoche content increased, and no significant increase in protein, lipids, and moisture content was observed by adding huitlacoche flour to tortilla chips [70]. Furthermore, it has been reported that adding huitlacoche powder can improve the physicochemical, rheological, and thermal properties of blue corn flour and “*masa*” and modify the color of blue corn flour and “*masa*”, changing it from a blue to a black color. However, in an industrial process, huitlacoche only makes up 9% of the total weight of the ingredients used in the formulation of blue corn flour and “*masa*” due to effect on the cohesiveness and adhesiveness of the resultant products [48].

Fettuccine pasta supplemented with huitlacoche powder (5 to 25% in weight) has shown a significant increase in dietary fiber (1.93 g/100 g), phenolic compounds (↑300%), and antioxidant activity (↑100%) content, in a huitlacoche concentration-dependent manner compared to the control paste (dietary fiber = 0.01 g/100 g); moreover, the addition of huitlacoche did not alter the technological properties (cooking time, cooking loss, water absorption, water solubility, swelling powder, ad density) of fettuccine pasta [8].

The use of the chlorogenic acid esterase (enzyme isolated from *U. maydis*) to make bakery products had positive softening effects. The addition of this enzyme improves the dough’s rheological parameters; moreover, the enzyme exhibited low thermostability, which is an advantage for baking. In this context, this enzyme could be a technological alternative to improve the taste and digestibility of diverse food products, mainly those rich in chlorogenic acid because their astringency characterizes this kind of product [122].

Functionalizing traditional and modern food products using huitlacoche flour is a technological alternative to promote the consumption of this fungal resource. It can be added to bakery and corn-based food products, increasing the dietary fiber and antioxidant molecules of foods that provide human health effects.

#### 3.4.3. Biocontrol Agent for Wine Production

Although *Brettanomyces bruxellenis*, a spoilage yeast, has a considerable impact on wine production, few tools are available to control its proliferation. In this regard, *U. maydis* CTC 1410 can produce a killer toxin (KP6-related toxin) that is effective against *B. bruxellenis* at low concentrations (400–2000 UA/mL) and acidic conditions (pH values from 3 to 4.5). This toxin is a small protein (encoded by dsRNA mycoviruses) that can be employed as a biological control strategy for wine production at the beginning of fermentation and aging [123].

#### 3.4.4. Antimicrobial Activity

A vast and mostly untapped source of bioactive compounds with potential biotechnological uses is found in fungus secondary metabolites [124]. The antimicrobial properties of *U. maydis* extracts or isolated compounds against bacteria, yeast, and molds have also been investigated. Glycolipids (by *U. maydis* FBD12) exhibited antimicrobial activity against *Staphylococcus aureus* and *Salmonella enterica* var. *Typhimurium* at low doses (MIC value of 0.01 to 0.04 mg/mL) after 24 h of exposure [106]. The antimicrobial effect of glycolipids is explained by an alteration of membrane permeability, promoting cell death. Furthermore, glycolipids (Ustilagic acid C and B) from *U. maydis* exhibited moderate antifungal activity (MIC values of 50 to 100 µg/mL) against *Aspergillus terreus* and *Candida albicans* [109]. Additionally, it has been reported that Mannosylerythritol lipids exhibited antimicrobial effects against *Bacillus subtilis* in a concentration-dependent response [108]. In this context, *U. maydis* could be a good source of antimicrobial compounds with potential pharmaceutical and food industry applications.

#### 3.4.5. Miscellaneous Applications

*U. maydis* is widely used in traditional medicine to treat diverse ailments; these beneficial effects are attributed to compounds with biological activities. Therefore, *U. maydis* has been explored for potential pharmaceutical applications, as discussed below.

Ustilipids are compounds extracted from the mycelium of *U. maydis* that exhibit dopaminergic properties; they act as antagonists of dopamine D_2_ and D_3_ receptors, which may be associated with the fatty acid profile of these compounds that include oleic, linoleic, stearic, palmitic, myristic, capric, caprylic, and caproic acids, indicating the pharmacological potential of ustilipids in the treatment of some neuroleptic diseases [95].

Additionally, it has been reported that Ustilagol compounds isolated from *U. maydis* MZ496986 exert antiplatelet and anti-inflammatory properties. Ustilagol G exhibited strong antiplatelet aggregation (IC_50_ = 16.5 µM) in U46619-stimulated human platelets, similar to that observed with aspirin (IC_50_ = 62.8 µM). Moreover, Ustilagol C and Ustilagol E showed anti-inflammatory properties in an LPS-induced macrophage RAW 264.7 model, associated with the structural configuration of these compounds and the ubication of methoxy groups at C-1, reducing NF-κB; however, the effect was in a dose-dependent response. These compounds could be explored as an alternative for neurodegenerative diseases [13].

The antimutagenic activity of *U. maydis* methanolic extracts from raw and cooked samples using a *Salmonella typhimurium* histidine reversion (his^−^ to his^+^) has been evaluated. These extracts showed antimutagenic activity ranging from 41 to 76%; however, these effects depended on maize genotype, the stage of maturity, and the cooking method. The antimutagenic activity of *U. maydis* extracts appears to be acceptable [69].

The antitumoral properties of Ustilagomaydisin A–C on multi-drug-resistant tumors has also been explored. These compounds are purine-derived compounds isolated from ethanolic extracts of *U. maydis*. These compounds have been shown to be weakly active against K562/A02 human leukemia cells compared to the drug verapamil [93].

The potential use of *U. maydis* as a platform to produce oral vaccines for cholera toxins has also been studied [125]. For this, huitlacoche has undergone genetic engineering to examine the expression and immunogenicity of the cholera toxin’s B subunit (CTB, secreted by *Vibrio cholerae*). Then, 12-week-old female BALB/c mice previously immunized with the oral vaccine based on recombinant CTB protein were challenged with the cholera toxin. Mice given an oral dose of CTB produced from huitlacoche exhibited substantial humoral responses linked with protection from the cholera toxin challenge. Furthermore, the oral vaccine maintained its immunogenetic activity after one year of storage at room temperature without reduction in CTB at 50 °C for 2 h, indicating its stability and immunogen effectivity [126].

According to these data, various extracts of isolated compounds of *U. maydis* can exert dopaminergic, antiplatelet, anti-inflammatory, antimutagenic, and antitumoral effects, which warrant additional research regarding the specific mechanisms of action and possible applications. Moreover, *U. maydis* could be an effective, safe, and low-cost platform for developing oral vaccines.

#### 3.4.6. Synthesis of Inorganic Nanoparticles

In recent years, the green synthesis of inorganic nanoparticles has exhibited a growing trend because it is an easy, rapid, eco-friendly, and low-cost alternative compared to the traditional chemical routes. In this context, natural resources able to act as reducing and stabilizing agents are needed during synthesis. Cortés-Camargo et al. [127] recently used huitlacoche aqueous extract as a reducing and stabilizing agent for synthetizing silver (Ag) nanoparticles. They found that the aqueous extract of huitlacoche is a good reducing agent (from AgNO_3_ to Ag) due to the high content of amino acids. Nonetheless, it acts as a stabilizing agent (zeta potential of −10.75 mV), avoiding the agglomeration and sedimentation of Ag nanoparticles, which exhibited quasi-spherical shapes within 100 to 5000 nm. Similarly, Bakur et al. [128] synthesized Au nanoparticles using mannosylerythritol lipid (MEL, obtained from *U. maydis* fermentation) as a reducing and capping agent under alkaline conditions. They found that MELs could reduce HAuCl_4_ to obtain Au nanoparticles with spherical shapes, associated with their biosurfactant properties. These materials exhibited in vitro antimicrobial, anticancer, and antioxidant activities. According to these data, *U. maydis* (extracts or compounds) could be a technological alternative as a reducing/stabilizing agent to the synthesis of inorganic nanoparticles with biological activities by green synthesis methods.

#### 3.4.7. Bioremediation

Every day, increasinh numbers of pollutants are released into all kinds of open waters; therefore, water treatment has received a lot of attention. In this context, *U. maydis* has been investigated as a biological alternative for heavy metal removal. Serrano-Gómez et al. [129] reported that the modification of *U. maydis* with formaldehyde can facilitate Cr(VI) biosorption from aqueous solutions in a pH-dependent manner. According to the authors, the adsorption of Cr(VI) is achieved in acidic conditions by electrostatic binding between the negative charge of the anion Cr(VI) and the positive charge of NH_3_^+^ groups (after the protonation of the -NH_2_ group), which are associated with the amino acids of *U. maydis*. Additionally, it has been reported that the biosorption of heavy metals such as Cr(III), Cd(II), Cu(II), Zn(II), and Ni(II) was successfully assessed using chitosan microcapsules functionalized with immobilized microfungal spores of *U. maydis*. However, the initial metal ion concentration, temperature, time, pH, and amount of sorbent all affect how effective this hybrid material is [130]. According to these data, *U. maydis* could be used as a potential bioremediation agent to remove heavy metals from aqueous media.

#### 3.4.8. Other Investigated Applications

Merkevičiūte-Venslovė et al. [49] evaluated the effect of *U. maydis* on the quality (nutritive value and aerobic deterioration) of maize silage. They prepared 50% and 100% silage infected with *U. maydis*. After 90 days, silage produced from maize that was 50% and 100% *U. maydis*-infected exhibited poor quality (↓protein and fiber content), with decreased dry matter loss (↓1.2% and 8%, respectively) and decreased starch (↓12.5% and 33%, respectively) content compared to *U. maydis* free silage. They concluded that *U. maydis* negatively affects the quality of maize silage, probably due to the influence of this fungal resource with aerobic bacteria that promotes the fermentative process. On the other hand, they also mentioned that the silage that was 50% and 100% *U. maydis*-infected did not promote any adverse effect on livestock health and production.

## 4. Toxicity and Safety Use of Huitlacoche

Huitlacoche has been investigated to evaluate its toxicity, and in general no harmful substances have been reported [49,83,131]. However, there are scarce literature studies that have reported toxicological effects of huitlacoche consumption.

One of the first reports on toxicological effects of *U. maydis* in humans was published in 1946 by Moore et al. [132]. They informed that an adult farmer died of chronic leptomeningitis “possibly associated” with *U. maydis*; however, no fungus cultures were isolated and identified during the autopsy. Furthermore, there is one case report of central line-related bloodstream infection caused by *U. maydis* consumption in a 64-year-old man with stage IV colon adenocarcinoma, followed by numerous chemotherapy regimens [133]. Additionally, some *Ustilago* species have been associated with an unusual case of peritonitis in a 3-year-old male that suffered from hypertension, end-stage renal disease, and chronic peritoneal dialysis; the patient “… *denied consumption of huitlacoche*…” but mentioned that his diet is based on traditional Mexican foods such as corn tortillas, which “*could contain huitlacoche spores*” [134]. It must be noted that the consumption of huitlacoche may promote some allergic reactions, similar to other foods, mainly sensitizing patients to rhinitis and asthma [135,136,137]. Moreover, in the reported clinical cases, patients showed a compromised immune system that could react to *Ustilago maydis* [133,134].

Additionally, there are some reports on the toxicological effects of *U. maydis* on rats; however, these studies were closely related to mycotoxins. Pepeljnjak et al. [138] evaluated the toxic effects of *U. maydis* and fumonisin B_1_ in female Fisher rats and reported neurotoxicity; however, the negative effects were associated with the fumonisin B_1_ and mentioned that mycotoxin can be found in huitlacoche. Recently, the presence of mycotoxins (aflatoxin, fumonisin, deoxynivalenol, and cyclopiazonic acid) was reported in commercial fresh and canned huitlacoche [139]. According to Pataky [57], fungal species such as *Fusarium*, *Aspergillus*, *Penicillium*, and *Mucor* can colonize very mature corn galls and produce mycotoxins, making them harmful if eaten. These results suggested that various mycotoxigenic fungi from the field can contaminate huitlacoche, as has happened with corn. In this context, some strategies and official standards are needed to avoid, prevent, or treat fungal/mycotoxin contamination to guarantee the safe production and/or consumption of huitlacoche [83].

On the other hand, this fungal resource has been eaten for centuries in Mexico, indicating that huitlacoche is a safe edible fungus [94]. Nonetheless, its consumption has recently gained international attention as gourmet food [4,84]. Interestingly, huitlacoche has been listed as an edible fungus in Switzerland [102]. Unfortunately, it has not been generally recognized as safe (GRAS) by the European Food Safety Authority (EFSA) or Food and Drug Administration (FDA) yet [83], limiting its acceptance in many countries around the world [84]. Therefore, developing suitable protocols to produce huitlacoche is required, and further studies are still needed to validate the non-toxicity of huitlacoche in order to obtain the standard of international recognition as a GRAS fungus.

## 5. Sustainable Development and Food Security

**Figure 9:**
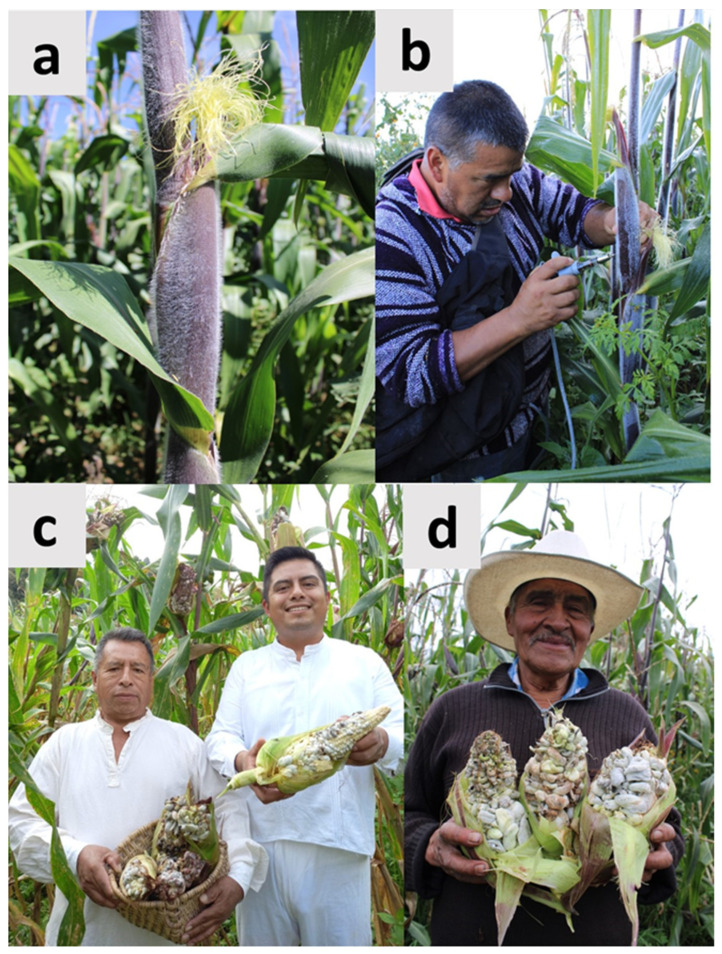
Cultivation of huitlacoche with a simple, efficient and cheap technology. (**a**) Stage at which the inoculation of corn should be made in young corn called “*jilotes*”; (**b**) Peasant of nahua origin injecting the huitlacoche inoculum with a special syringe; (**c**) Harvest of cultivated huitlacoche by Mazatec People, in Oaxaca in Southern Mexico; (**d**) Big huitlacoche corns produced by the inoculation of the inoculum, in San Felipe Hidalgo, Tlaxcala, in Central Mexico, as a consequence of the application of a technology generated during the last decade.

Mexico is the center of domestication of corn, one of the most important foods worldwide; currently, the country has 64 recognized strains, called landraces, and over 21,000 regionally adapted varieties. Over two-thirds of Mexican corn farmers still save their own seeds and plant native strains. However, due to economic, social, and cultural aspects, the preservation of these breeds, which constitute an invaluable genetic resource generated over thousands of years of domestication, is at extinction risk. One of the main reasons that explains this scenario is the extensive planting of hybrid varieties, which generate greater economic gains than native breeds, and the indiscriminate use of pesticides and fertilizers to increase the production. In contrast, in the case of native maize, its productivity is usually lower because agroecological techniques are frequently used in its production, such as the polyculture system called *milpa*, which leads to soil conservation; this is not like the massive production systems used for hybrid maize that lead to desertification in the long term. In this scenario, the authors of the present contribution have initiated a program for the cultivation of huitlacoche in native maize in Central and Southeastern Mexico (Figure 9). The results have shown that the cultivation of this fungus in sections of the producers’ plots has encouraged the conservation of native maize breeds. The reasons for the success of this program are: (i) The high cost of huitlacoche, compared to corn. In general terms, the net gains can be 20 to 50 times higher when huitlacoche is grown compared to corn; (ii) The huitlacoche is culturally highly appreciated as food in Central and Southeastern Mexico, and its natural production, which was always low (around 1% of infected plants in the field cultivation plots), has decreased dramatically in the last decade, becoming almost zero in 2022 due to global change and the associated changes in rainfall patterns and increases in temperature; (iii) During the last decade, the authors of the present contribution have developed a low-cost, high-efficiency technology and simple methods for the inoculation of maize plants, easily adopted by maize producers; and (iv) There is a whole mycogastronomic culture around this fungus in Central and Southeastern Mexico. This ranges from the massive consumption of traditional Mexican dishes to the preparation of gourmet dishes, which greatly facilitates its commercialization. Currently, a transdisciplinary project of high-level scientific research and social incidence funded by the National Council of Science and Technology of Mexico (CONACyT), led by one of the corresponding authors of this contribution (JPM), aims to promote the cultivation of huitlacoche in native varieties of Mexico, tending to their conservation and taking into account their financial viability and the favorable sociocultural conditions existing in Central and Southeastern Mexico. The cultivation of huitlacoche constitutes a strategy linked to food security and sustainable development, and it is aligned with the Sustainable Development Goals (SDG) of the UN2023 [92] for the following reasons: (i) The huitlacoche is a food with high nutritional value that can contribute to reducing hunger (SDG 1: No poverty and SDG 2: Zero hunger); (ii) Huitlacoche is a food that also contributes to human health and has been used in traditional Mexican medicine for centuries, as mentioned above (SDG3: Good Health and Well-being); and (iii) If women’s cooperatives are established, the empowerment of peasant women is feasible, given that if surpluses produced are sold to those in domestic markets, it is feasible to industrialize this food by producing canned, dehydrated, or brined huitlacoche, which extends its shelf life and increases the financial gains produced by its cultivation (SDG 5: Gender Equality, SDG 8: Decent work and Economic Growth and SDG 10: Reduced Inequalities).

## 6. Conclusions

In summary, huitlacoche is one of the most important edible fungi with biocultural significance in Mexico; currently, it is traditionally consumed by diverse ethnic groups, and it is also used in a wide variety of food dishes throughout the country. Moreover, this fungal resource is a crop with agro-alimentary importance and is a functional food with commercial value. Evidence shows that huitlacoche is a valuable food source with high nutritional value (in terms of protein, dietary fiber, fatty acids, minerals, and vitamin contents) and bioactive compounds such as polyphenols, flavonoids, carotenoids, phytosterols, purine-derived, and terpenoids, with health-enhancing properties. Previously, it has been demonstrated that these compounds have relevant biological activities, including diverse functions such as antioxidant, antimicrobial, anti-inflammatory, antimutagenic, dopaminergic and antiplatelet effects. Additionally, huitlacoche fungus contains compounds that are stabilizing and capping agents for inorganic nanoparticle synthesis, involved in the remotion of heavy metals from aqueous media, biocontrol agents for wine production, and also have industrial potential, e.g., by producing biosurfactant compounds and enzymes.

Additionally, it is feasible that the cultivation of huitlacoche may contribute to food security, sustainable development, food diversification, human nutrition and health, economic development, conservation of biocultural heritage, women’s empowerment, and hunger mitigation, as has been pointed out for other groups of fungi (e.g., ectomycorrhizal mushrooms). However, in order to achieve this, it is necessary to develop strategic alliances (ODS17 Partnerships for the goals) involving policy makers, entrepreneurs, scientists, and different social sectors, with emphasis on rural population.

## Figures and Tables

**Table 1:** Traditional names assigned to *Ustilago maydis* by different Mexican ethnic groups.

Ethnic Group	State	Common Name in Traditional Language	English Translation	Ref.
Maya	Yucatan	*Ta’ chak, ta’ chak ixia*	Excrement of the Maya God Chak in the corn	[16]
Totonac	Veracruz	*Xanat kuxi*	Corn flower	[17]
Tzeltal	Chiapas	*Lu’, sakil ti’bal*	Donkey testicles	[18]
Tzeltal	Chiapas	*Slu ‘il ixim*	Corn fungus	[19]
Tzotzil	Chiapas	*Stok’al ixim, sjo’jal ajan*	Corn cloud storm, Corn fungus	[20]
Tzotzil	Chiapas	*Tok*	Cloud	[21]
Tzotzil	Chiapas	*Xu’ixim, chikin te*	Milk from the cornfield, Stick ear	[18]
Lacandón	Chiapas	*Ta’ urim nar*	Corn fungus	[21]
Tojolabal	Chiapas	*Chikin chu’*	Corn fungus	[22]
Mazatec	Oaxaca	*Tohíjé*	Ball of young corn	[23]
Mixtec	Oaxaca	*T* *ɨ* *k* *á* *maa*	Bad grasshooper	[24]
Mixtec	Oaxaca	*Tikayá*	Round bleached	[25]
Mixtec	Guerrero	*Xi’i itu’u*	Corn fungus	Inedit
Chinantec	Oaxaca	*Dséc cui*	Son or shoot of the cornfield	[26]
Zapotec	Oaxaca	*Bia’huí’*	Moldy guava	[27]
Zapotec	Oaxaca	*Mey guiel*	Corn fungus	[28]
Zapotec	Oaxaca	*Xobdam*	Owl corn	[29]
Zapotec	Oaxaca	*Bzodlan*	Ear fungus	[30]
Zapotec	Oaxaca	*Měy-guiêl-do*	Corn tassel fungus	[31]
Otomí	Estado de México	*Kjú tha*	Lost ear	[32]
Nahua	Tlaxcala	*Cuitlacoche*	Excrement	[33]
Náhuatl	Hidalgo	*Kjod kja*	Corn fungus	[34]
Tepéhua	Hidalgo	*Búas*	Excrement	[35]
Otomí	Hidalgo	*Kjo thä, kjo ra mancha*	Corn fungus, cornfield fungus, ear fungus	[36]
Pjiekakjoo	Estado de México	*Nchjo pa*	Cornfield fungus	Inedit
Purépecha	Michoacan	*Terékua poxi*	Corn fungus	[37]
Nahua	Puebla	*Tacatzazamazlat*	Excrement fungus	[38]
Wixaritari	Jalisco	*Ku’u*	Corn fungus	[39]
Tarahumara	Chihuahua	*Weko wiwara*	Corn fungus	[36]
Rarámuri	Chihuahua	*Witáchori*	Excrement	[40]
Tepehuan	Durango	*Jaroi* o *jurá*	Heart	[36]
Pima	Sonora	*Nanha*	Corn smut	[40]

**Table 2:** Nutritional composition and energy value of huitlacoche.

Parameter	References				
	[6]	[8]	[15]	[69]	[65]
Moisture (g/100 g)	26.81	^§^ 8.55	90	92–96	80–86
Ash (g/100 g)	3.37	5.66	ND	4–8	3.8–5.3
Protein (g/100 g)	3.27	8.08	12	12–14	12.4
Total fat (g/100 g)	0.73	1.14	1.8	4–6	2.9
Carbohydrates (g/100 g)	57.2	64.43	45	72–86	54–65
Total fiber (g/100 g)	* 8.61	* 12.14	ND	^¥^ 39–60	^¥^ 47–49
Soluble dietary fiber (g/100 g)	ND	ND	ND	9–29	ND
Insoluble dietary fiber (g/100 g)	ND	ND	ND	22–51	ND
β-glucans (mg/100 g)	ND	ND	ND	20–120	ND

* Crude fiber; ^¥^ Total dietary fiber; ^§^ dry basis; ND = Not determined.

**Table 3:** Amino acid, fatty acid, sugars, and mineral contents of huitlacoche.

Amino Acids	Content (mg/g)	Fatty Acids	Content (%)	Sugars	Content (mg/g)	Minerals	Content (mg/g)
Lysine	3.21	Oleic acid	42.49	Total free sugars	56–267	Phosphorous	0.342
Glycine	2.44	Linoleic acid	26.97	Glucose	53–231	Magnesium	0.262
Leucine	2.24	Palmitic acid	14.79	Fructose	19–138	Calcium	0.018
Glutamic acid	1.90	11-Eicosenoic acid	4.39	Galactose	0.2–3.5	Sodium	0.012
Aspartic acid	1.80	Stearic acid	3.94	Arabinose	0.2–3.3	Iron	0.0028
Valine	1.46	Arachidic acid	2.86	Mannose	0–1.8	Zinc	0.0025
Isoleucine	1.32	Palmitoleic acid	2.10	Xylose	0.2	Manganese	0.0019
Phenylalanine	1.16	Linolenic acid	0.84				
Alanine	1.05	Pentadecanoic acid	0.67				
Serine	1.02	Margaric acid	0.51				
Tyrosine	1.00	Myristic acid	0.44				
Proline	0.75	Behenic	* 2.4–5.9				
Threonine	0.62	Lignoceric	* 1.2–2.7				
Methionine	0.15	Ergosterol	* 20–97				
Ornithine	0.08						
Tryptophane	0.05						
References	[89]		[15,63]		[65]		[15]

* numbers expressed in mg per g.

**Table 4:** Mycochemical content in huitlacoche.

Bioactive Compounds	Content	Ref.
**Phenolic compounds**		
Total phenolic compounds (mg GAE/100 g)	11–1394	[6,8,15,64,65]
Condensed tannins (mg Eq Catechin/100 g)	32.2–310	[65]
Chlorogenic acid (µg/g)	15.94	[64]
Methyl-gallate (µg/g)	4.19	[64]
Epicatechin (µg/g)	3.16	[64]
Ferulic acid (µg/g)	358	[63]
Gallic acid (µg/g)	0.4–1.5	[63,64]
Caffeic acid (µg/g)	11.2	[63]
Protocatechuic acid (µg/g)	0.00093	[64]
*o-*coumaric acid (µg/g)	5	[63]
*p-*coumaric acid(µg/g)	12	[63]
Sinapic acid (µg/g)	36	[63]
Syringic acid (µg/g)	0.0158	[64]
4-Hydroxybenzoic acid (µg/g)	0.0174	[64]
**Flavonoids**		
Total flavonoid (mg Catechin/kg)	28.51	[15]
Anthocyanins (mg/kg cianidin-3-glucoside)	89.8–226.3	[65]
Rutin (µg/g)	5	[63]
Catechin (µg/g)	10–11.42	[63,64]
Quercetin (µg/g)	33	[63]
Naringenin (µg/g)	14.1	[63]
**Carotenoids (µg/g)**	3.05	[64]
β-Carotene (µg/g)	15	[85]
β-Cryptoxanthin (µg/g)	1.13	[64]
Phytofluene (µg/g)	0.40	[64]
Lutein (µg/g)	0.31	[64]
Zeaxanthin (µg/g)	0.31	[64]
Luteoxanthin (µg/g)	0.63	[64]
**Phytosterols**		
Ergosterol (µg/g)	3.24–4.19	[64]
Campesterol-3-β-glucoside (µg/g)	8.25–12.94	[64]
Δ7-avenasterol (µg/g)	3.83–5.81	[64]
Δ7-estigmasterol (µg/g)	4.25–5.92	[64]
**Other compounds**		
Ustilagol A	Identified	[13]
Ustilagol B	Identified	[13]
Ustilagol C	Identified	[13]
Ustilagol D	Identified	[13]
Ustilagol E	Identified	[13]
Ustilagol F	Identified	[13]
Ustilagomaydisin A	Identified	[93]
Ustilagomaydisin B	Identified	[93]
Ustilagomaydisin C	Identified	[93]
Sesquiterpenoids	Identified	[94]
Ustilipid A	NI	[95]
Ustilipid B	NI	[95]
Ustilipid C	NI	[95]
Ergothioneine (µmol/g)	5.4	[96]

GAE = Gallic acid equivalents; Eq = Equivalent; NI = No information.

**Table 5:** Antioxidant capacity of huitlacoche extracts.

Bioactive Extracts or Compounds	Extraction Method	Method of Antioxidant Capacity				Reference
		ABTS•	DPPH•	FRAP	ORAC	
Hydroethanolic extract	Maceration	45.26 ^a^	13.16 ^a^	ND	ND	[6]
Hydroethanolic extract	UAE	26.45 ^a^	22.5 ^a^	ND	ND	[6]
Methanolic extract	Stirring	ND	56–74 ^b^	ND	ND	[65]
Methanol-Water	Shaking	ND	186.44 ^c^	ND	ND	[15]
Ethanolic extract	Magnetic stirring	200–312 ^d^	30–165 ^d^	117–215 ^d^	ND	[64]
Methanol-Water	Shaking	1652.42 ^e^	9.50 ^e^	64.8 ^e^	ND	[8]
Methanolic extract	Shaking	ND	ND	ND	41–76 ^e^	[63]

UAE = Ultrasound-Assisted Extraction; ORAC = Oxygen radical absorbance capacity; ^a^ µmol of trolox equivalents (TE)/mL; ^b^ Percentage of radical inhibition; ^c^ IC_50_, mg/mg DPPH; ^d^ mmol TE/mL; ^e^ µmol TE/g; ND = Not determined.

## Data Availability

Not applicable.
